# Performance of Optical Structural Vibration Monitoring Systems in Experimental Modal Analysis

**DOI:** 10.3390/s21041239

**Published:** 2021-02-10

**Authors:** Maksat Kalybek, Mateusz Bocian, Nikolaos Nikitas

**Affiliations:** 1 School of Engineering, University of Leicester, Leicester LE1 7RH, UK; m.bocian@leicester.ac.uk; 2 Dynamics, Vibration & Acoustics Laboratory, University of Leicester, Leicester LE1 7RH, UK; 3 School of Civil Engineering, University of Leeds, Leeds LS2 9JT, UK; n.nikitas@leeds.ac.uk

**Keywords:** optical systems, structural health monitoring, modal testing, vibration measurement, experimental modal analysis

## Abstract

Image-based optical vibration measurement is an attractive alternative to the conventional measurement of structural dynamics predominantly relying on accelerometry. Although various optical vibration monitoring systems are now readily available, their performance is currently not well defined, especially in the context of experimental modal analysis. To this end, this study provides some of the first evidence of the capability of optical vibration monitoring systems in modal identification using input–output measurements. A comparative study is conducted on a scaled model of a 3D building frame set in a laboratory environment. The dynamic response of the model to an impulse excitation from an instrumented hammer, and an initial displacement, is measured by means of five optical motion capture systems. These include commercial and open-source systems based on laser Doppler velocimetry, fiducial markers and marker-less pattern recognition. The performance of these systems is analysed against the data obtained with a set of high-precision accelerometers. It is shown that the modal parameters identified from each system are not always equivalent, and that each system has limitations inherent to its design. Informed by these findings, a guidance for the deployment of the considered optical motion capture systems is given, aiding in their choice and implementation for structural vibration monitoring.

## 1. Introduction

Optical motion capture systems (MCS) are becoming increasingly popular in structural vibration monitoring [1,2,3]. Their main advantage lies in the remote operation hence avoidance of the deployment of cabling system associated with conventional motion capture systems, most often relying on accelerometry. Although optical MCS derive the most accurate results tracking a single point only, continuous improvement in resolution of all sorts of digital cameras has enabled vibration tests to be performed in which multiple points are being tracked simultaneously. For example, Park et al. [4] presented results from tests utilising motion capture system (MCS) based on three cameras measuring 3D structural displacements in a laboratory environment. The process required installing multiple light-reflective markers on the structure, each seen by at least two cameras at any time. A number of studies explored the capabilities of single-camera systems. For example, Brownjohn et al. [5] and Luo et al. [6] used a single high speed camera coupled with custom software for tracking multiple points on bridges in situ. Patil et al. [7] proposed a method of stitching together mode shapes from data obtained from video footage of a pair of cameras roving around a structure for 3D measurements. As for the equipment, all these studies used off-the-shelf commercial products dedicated to motion capture.

Optical MCS could become a cost-effective solution for structural vibration monitoring, particularly when direct displacement rather than acceleration is sought. However, off-the-shelf commercial products from the leading suppliers carry a cost of tens of thousands GBP, which may be prohibitive for many potential users. Undoubtedly, these solutions are quite refined in terms of data processing capabilities and user-friendly interfaces. However, it remains to be determined whether simpler solutions relying on consumer-grade hardware and well documented data processing algorithms, often available in open-source format, can match their performance, or at least offer enough measurement fidelity to be considered viable alternative solutions. The results of recent investigations are encouraging in this respect. For example, Kromanis et al. [8] compared a number of modern smartphones against various cameras by coupling them with various image processing algorithms. By measuring deformations of structural elements caused by static, quasi-static and dynamic loads in the lab, they demonstrated the feasibility of such low-cost systems for structural health monitoring (SHM). A number of studies explored the capabilities of smartphone cameras in capturing the vibration and displacement of structures in situ [9,10], whilst other studies used action cameras for the same purpose. For example, Lydon et al. [11] explored a feature-based template matching technique [12] by tracking the displacement of a single point on two structures using Go-Pro camera. Xu et al. [13] conducted a similar study, but using area-based template matching technique [12] for tracking multiple points. A few attempts were also made to use other types of optical MCS, such as depth sensing cameras (RGB-D) [14] and video cameras [15,16], while an exhaustive review on relevant advances can be found elsewhere [3,17]. However, none of the studies provided a full dynamic characterisation of the tested structures, which is the ultimate goal of modal analysis [18].

A continuing effort is being made on developing advanced modal identification techniques based on operational modal analysis (OMA), in which the excitation force comes from either environmental and/or man-made sources, see e.g., the hybrid OMAX [19], associated with the operational regime of the investigated structure [20]. However, while pure OMA can provide information about the modal frequencies, damping, and mode shapes, it cannot intrinsically scale the mode shapes since it relies on response measurement only while making rather specific assumptions as to the nature of the excitation input [21]. Although a number of approaches has been proposed in the last two decades to overcome this limitation [22], their application requires either various types of interventions on the structure or its numerical model [23]. The former approach is realised by introducing a local change in the mass or stiffness of the structure, or an auxiliary mechanical system. Therefore, it carries a similarity with experimental modal analysis (EMA) in which an extraneous device is employed—here a force source, enabling direct scaling of the mode shapes with modal mass. The latter approach relies on a highly reliable finite element model, which is notoriously difficult to obtain even for seemingly simple civil structures. Consequently, EMA-based practices are still the gold standard in full dynamic characterisation of structures.

Although studies on full-scale structures in situ are the ultimate reason for interest in optical MCS within civil engineering community, laboratory-based experiments allow the MCS performance to be examined in a controlled environment while excluding any environmental effects which can debilitate the measurement accuracy. Such studies generally involve using simple structural elements, most often beams or columns [16], or custom-built downscaled models of simple structures, such as few-storeys high models of building frames [4,24,25,26,27]. The latter approach, with a more demanding variant of a 3D rather than a planar frame, is adopted in this study.

To the best of the authors’ knowledge, this is the first study aiming to assess the performance of various optical structural vibration monitoring systems in the context of EMA. Six instrumentation systems, including five optical MCS, are deployed in a laboratory environment to measure the response of a scaled frame of a 3D building to the impulse excitation from an instrumented hammer and initial displacement. A complete set of modal properties is used for benchmarking, including natural frequencies, damping ratios, mode shapes and modal (generalised) masses. The damping ratios and in particular modal masses seem to have never appeared in alike comparisons involving image-based optical MCS. The rest of the paper is organised as follows. Section 2 presents the tested structure, briefly introduces instrumentation systems used in this study, describes experimental protocols and outlines data analysis procedures. The results are presented and discussed in Section 3. This includes the results of pose reconstruction assessment of ArUco markers in Section 3.1, modal identification in Section 3.2 and initial displacement response in Section 3.3. The conclusions are presented in Section 4.

## 2. Methodology

### 2.1. Tested Structure

**Figure 1:**
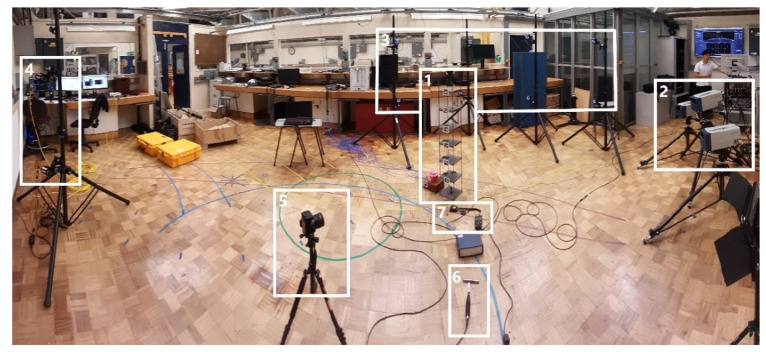
The layout of instrumentation systems in the Dynamics, Vibration, and Acoustics Laboratory at the University of Leicester: 1—Steel frame with accelerometers attached; 2—Polytec laser Doppler vibrometer; 3—OptiTrack; 4—Imetrum; 5—Consumer-grade camera (CGC); 6—instrumented hammer; 7—lux meter.

**Figure 2:**
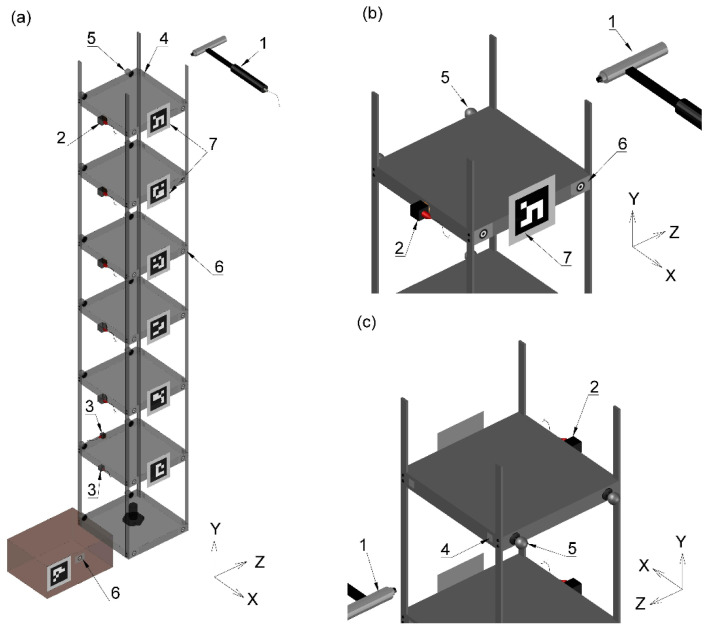
(**a**) Steel frame and (**b**,**c**) its details showing the positioning of sensors and markers around the top plate. 1—instrumented hammer; 2—triaxial accelerometer; 3—single axis accelerometer; 4—light reflective fiducial marker for laser Doppler vibrometer (LDV); 5—spherical reflective marker for OptiTrack; 6—Imetrum marker, 7—ArUco marker.

A simple steel frame shown in Figure 1 was designed and built at the University of Leicester, UK. SAP2000 version 20 structural analysis package was used in the specification of structural components such as to tune natural frequencies of the frame to those representative of full-scale civil structures such as short-span bridges and medium-rise buildings. The frame has six plates above the bottom plate centrally bolted onto the floor, each 20 mm thick and cut to fit within 180 × 180 mm outline, joined by four columns recessed into the plates’ corners. Mild steel with density 7850 kg·m^−3^ was used throughout. The distance between centroids of each two consecutive plates is 200 mm, except for the distance between plate 4 and 5 counting from the bottom, which is 180 mm. The columns have rectangular cross section of 10 × 3 mm and are connected to each plate using two screws arranged one above the other. The structure is rather lively, i.e., after providing a hit to one of the plates the structure can be seen with naked eye to vibrate for a prolonged time, indicating low damping. The structure together with its coordinate axes is shown in Figure 2. The movement in *z*-axis was of particular interest since it is associated with the weak (in terms of resistance of the section to bending/rotation) axis of the columns.

### 2.2. Instrumentation

A set of conventional constant current accelerometers and five optical motion capture systems, including two systems relying on images recorded with a consumer-grade camera (CGC), were used in this study. The basic specification of these systems is given in Table 1. An instrumented hammer was used to provide force excitation and a lux meter was used to measure light intensity during the tests. A brief description of these instrumentation systems is given in Section 2.2.1, Section 2.2.2, Section 2.2.3, Section 2.2.4, Section 2.2.5, Section 2.2.6 and Section 2.2.7. Figure 2 presents the layout of accelerometers and fiducial markers attached to the frame, used in conjunction with optical MCS, and the point of application of excitation force with the instrumented hammer, which is the centroid of the side face of the top plate. This point was chosen with an intention not to mobilise strongly torsional modes which could distort the results of modal analysis from instrumentation systems which were not inherently capable of 3D measurements. It can be seen in Figure 2 that the accelerometers and markers cover all side faces of the plates above the bottom plate. The layout of the instrumentation and markers was optimised such as to obtain maximum quality measurements in *z*-axis. For clarity of presentation, the operational principles behind the different fiducial markers are explained in Section 2.3. Basic specification of the MCS used in this study is given in Table 1.

#### 2.2.1. Accelerometers

Acceleration in x- and *z*-axis (see Figure 2) was measured with five triaxial accelerometers (PCB 356 A16, USA) mounted onto plates 3–7, counting from the base (i.e., bottom) plate, and two single axis accelerometers (PCB 333 B30, USA) mounted onto plate 2. The positions of accelerometers measuring the response in *z*-axis were collinear since they were located at the centroids of the plates’ faces on the same side of the frame, as shown in Figure 2a.

#### 2.2.2. Imetrum

Imetrum [28] is a commercial-grade MCS used for structural testing in a variety of applications in a laboratory and outdoor environment [5,29]. The main asset of the system is the Video Gauge software, developed based on the study of Macdonald et al. [30], which uses Digital Image Correlation (DIC) technique coupled with various prediction and subpixel refinement techniques. The setup used in this study consisted of a dedicated PC with Video Gauge software package, two synchronised cameras with 2 megapixel (MP) resolution equipped with 25 mm camera lenses, recording at 50 frames per second (fps) and mounted on a single tripod approximately 1.2 m apart. To perform 3D measurements, the calibration process includes specifying the inter camera distances and angles, and predefining some known distances within the captured image. 2D markers consisting of slightly blurred black and white concentric circles printed on white matte vinyl stickers were attached to the plates, as shown in Figure 2b, for improved tracking quality as the steel plate surface was found to be rather featureless at the scale of interest.

#### 2.2.3. OptiTrack

OptiTrack [31] is a commercial-grade MCS used predominantly for laboratory testing in biomechanics, robotics and virtual reality applications. The setup used in this study consisted of eight Prime 13 cameras with 1.3 MP resolution and sampling at 120 fps, mounted in pairs on four tripods, a network hub and Motive 2.1.1 motion capture software. The system detects and tracks the movement of spherical reflective markers which were attached to the plates, as shown in Figure 2. A 250 mm T-shaped wand and 200 mm square were used to calibrate the cameras and define the coordinate system aligned with that of the frame.

#### 2.2.4. Polytec Laser Doppler Vibrometer (LDV)

Polytec laser Doppler vibrometer (LDV) PSV-500-3D was used in this study. It consisted of three HeNe red laser scanning heads enabling 3D measurement, one of which includes video camera facilitating system set up. The scanning heads were positioned at a distance of about 3 m from the frame and, since they utilise the Doppler effect, they were pointing at the plates’ faces perpendicular to the *z*-axis at the angles close to 90 degrees to obtain reliable measurements of the structural response in *z*-axis. A proprietary PC with data acquisition board and PSV software was used during measurements. In order to perform 3D scan, all three laser heads were set up to measure one point of interest at a time. For better reflection of laser signals hence improved signal to noise ratio, 2D light reflective fiducial markers were attached to the plates of the structure as shown in Figure 2c.

#### 2.2.5. Consumer-Grade Camera (CGC)

Canon EOS 200D CGC coupled with 20 mm focal length lens, with maximum aperture f/2.8, was used in this study, set to operate in a fixed focus mode. The video footage recorded with CGC was used with two MCS, (i) ArUco system tracking feature-based fiducial markers and (ii) area-based template matching system hereafter referred to as template matching and abbreviated by TM. The data processing was implemented in a custom application written in C++ programming language, referring to open source OpenCV library of computer vision functions [32]. Sub-pixel refinement was used in both CGC-based MCS.

##### Feature-Based Template Matching (ArUco)

ArUco system, presented in Garrido-Jurado et al. [33], has so far been mainly used for pose estimation in computer vision applications such as robot navigation and augmented reality. It is believed to be one of the most evolved tools for fiducial marker detection [34]. The markers are composed of a wide black border and an inner binary matrix which determines their unique IDs. Marker tracking is performed in two stages: (i) Marker detection and (ii) estimation of markers’ pose relative to the camera. During stage (i) the outer square box and the unique pattern of markers’ inner binary code are identified. This inner pattern allows robust detection of multiple markers in an image frame. During stage (ii) the position and rotation of camera related to each marker is identified through a full projection matrix [35]. This is a camera calibration process where camera intrinsic parameters are identified using the images of a chessboard and then the camera extrinsic parameters are identified through the known physical geometry of the markers. For this study, the movement of the markers attached to the steel frame was identified relative to their state in the first frame of the captured video.

##### Area-Based Template Matching

The video from CGC used with ArUco was also used in an area-based template matching system, which in various forms is now being widely used for SHM [2,36]. The area-based template matching relies on the identification of template patterns within captured images. The normalised version of sum of squared differences (NSSD) [32] method was used as a correlation criterion. For improved computational efficiency, a region of interest (ROI) was defined, reducing the area of images within which to search for the template patterns. An offline calibration with chessboard was performed to remove camera distortion effects. A planar homography matrix was determined through linking the user-defined planar coordinates of key points on the 2D structural coordinate system and the pixel coordinates of these points. The area-based template matching system, hereafter simply referred to as template matching, used the ArUco markers to aid in pattern recognition.

#### 2.2.6. Instrumented Hammer

**Figure 3:**
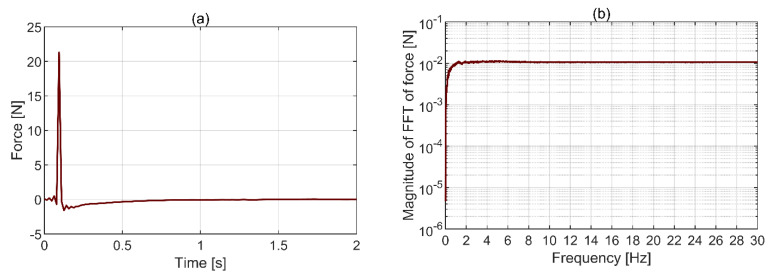
Hammer force in (**a**) time domain and (**b**) frequency domain.

PCB 086C01 instrumented hammer was used to provide excitation energy to the structure. A pre-trigger offset was set based on the force threshold to ensure the whole signal was contained within the measured window. The hammer tip was chosen such as to ensure there is enough excitation energy within the whole frequency range of interest. An example of the recorded hammer force in time and frequency domains is shown in Figure 3a,b. The time and frequency ranges were truncated to highlight the shape of the force pulse and the magnitude of the FFT of the force within the frequency range of interest, respectively.

The ripples on both sides of the pulse in Figure 3a are a consequence of applying anti-aliasing (low pass) filter on the force signal and indicate its frequency content goes beyond the limit set in data acquisition. This does not affect the quality of the analysis presented in this study [23]. It can be seen in Figure 3b that the force spectrum between 1.5 Hz and 30 Hz is flat hence there is energy in the frequency band of interest of 1.5 to 25 Hz. The lower frequency limit was chosen considering the lowest expected mode in *z*-axis of the structure, and the upper limit is dictated by the maximum sampling frequency of Imetrum. Further discussion on the adequacy of the chosen excitation method for extracting response characteristics is given in Section 3.2.

#### 2.2.7. Lux Meter

Chauvin Arnoux CA1110 lux meter was used to record light intensity during testing. The lux meter was positioned close to the frame, as shown in Figure 1, and sampled at 1 Hz. The expected measurement error stated by the manufacturer at the light intensity levels close to those recorded during the tests is below 1%. The mean and standard deviation of light intensity recorded during the tests was 686 lx and 5.8 lx, respectively. Therefore, the measurements from optics-based systems can be considered free from errors associated with the light intensity fluctuations. The recorded illuminance levels indicate the lab was well lit and represent recommended conditions for areas in which precision work is conducted [37].

### 2.3. Fiducial Markers

Different types of fiducial markers were used in conjunction with the optical instrumentation systems described in Section 2.2. In a broad sense, fiducial markers are reference objects deliberately set within the monitored system to facilitate recognition, localisation and tracking. They are often used in medicine, robotics, measurement and surveying, and X-Reality (or Cross-Reality)—a term encompassing a wide range of technology enabling realisation of virtual environments, typically in applications involving computer vision. In the context of structural vibration monitoring, light-reflective, light-emitting, and shape- and pattern-based fiducial markers were previously used to aid motion recognition using optical systems.

#### 2.3.1. Light-Reflective Markers

**Figure 4:**
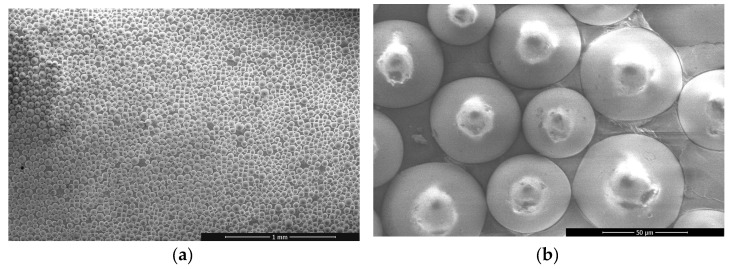
Light reflective marker under a scanning electron microscope with (**a**) 130 magnification and (**b**) 2000 magnification.

Light-reflective fiducial markers comprise of a flat or spherical object covered with retroreflective paint thus bouncing the light back towards the light source, hence enhancing marker visibility. This is typically achieved with tightly-spaced 20–90 µm diameter glass spheres having rear surface covered with highly reflective coating, as seen on pictures taken with a scanning electron microscope in Figure 4. For maximum efficiency, the light source is integrated with the optical measurement system, e.g., infrared LEDs surrounding lenses of OptiTrack cameras. Light-reflective fiducial markers have been used for measurements taken in a laboratory environment [38] and in the field [39]. In this study, light-reflective fiducial markers were used to facilitate the operation of OptiTrack system described in Section 2.2.3 and enhance the performance of LDV system described in Section 2.2.4.

#### 2.3.2. Light-Emitting Markers

Light-emitting fiducial markers produce light to reveal their location within captured images [40]. This approach is advocated to reduce the problem caused by insufficient and/or non-uniform structure illumination, e.g., caused by vapour interference [41]. Although markers of this type were not used in this study, they are mentioned here for completeness.

#### 2.3.3. Shape- and Pattern-Based Markers

Shape and pattern-based fiducial markers are graphical objects of predefined geometry set within captured images. Shapes are 2D components of relatively simple topological structure, e.g., dots or few-sided polygons, whilst patterns are 2D assemblies of shapes. The recognition of shapes typically involves edge detection, object fitting and/or centroid computation [42]. Patterns are usually used to enable marker pose estimation, i.e., the determination of its position and orientation in 2D or 3D space [33]. Pattern-based fiducial markers were used by ArUco and template matching systems described in Section 2.2.5, and aided in area-based recognition by Imetrum system described in Section 2.2.2.

### 2.4. ArUco Pose Accuracy Assessment

**Figure 5:**
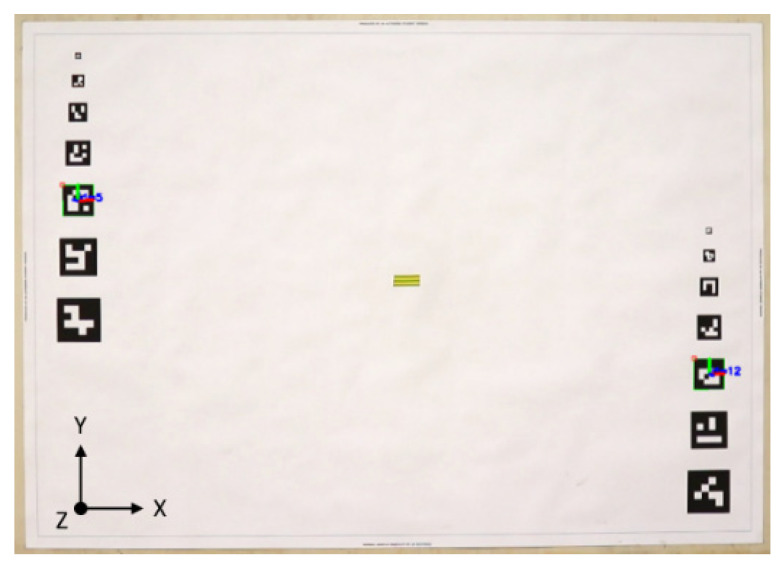
A screen shot of ArUco pose reconstruction accuracy test. Two markers of the same size are identified within the picture and overlaid with the local marker axes using ArUco proprietary OpenCV library.

Prior to the deployment of ArUco system on the structure it was necessary to establish the accuracy of the estimated pose. Furthermore, suitable marker size, camera to structure distance and angle of the camera relative to the structure (i.e., angle of incidence) had to be determined considering the camera and lens capabilities. To this end, two parallel columns of black and white ArUco square markers, varying in side size from 1 to 7 cm every 1 cm, were printed along the two short edges of an A0 sheet attached to an inch thick fibreboard clamped against a wall, as shown in Figure 5. All markers had unique patterns and were positioned at different height such that the planar coordinates of the centres of any two markers of the same size were different but the distances between them were the same. The longest distance between a pair of markers of corresponding size measured along *x*-axis shown in Figure 5 was representative of the longest distance between ArUco markers placed on the frame during modal and initial displacement tests. The CGC was placed at various distances (1.5 m to 4 m every 0.5 m) and facing angles (0 degrees, 15 degrees, and 30 degrees) to the board at the height corresponding to the middle of the sheet, represented by a yellow patch in the middle of Figure 5. A one-minute video was recorded for each combination of the camera arrangement. The results of ArUco pose accuracy assessment are presented in Section 3.1.

### 2.5. Modal Testing and Analysis

The centroid of the side face of the top plate of the structure was hit 8 times with the instrumented hammer in *z*-axis to obtain correspondingly 8 windows of 64 s duration, containing time-synchronised structural response data from accelerometers and input force from the hammer. The length of the window was chosen such as to ensure vibration amplitudes would decay to values below observable. However, since the signals from camera-based MCS contained significant amount of noise at low vibration levels, an exponential decay window was applied to the corresponding force and response signals in each widow during modal analysis. The artificial (i.e., numerical) damping was subsequently removed to obtain unbiased modal damping estimates. The acquisition of data by all optical systems was performed in parallel, apart from LDV since it cannot be used to obtain the structural response data at more than one location at the time. Therefore, a dedicated test was run for the purpose of modal testing with LDV. For each of the 14 locations measured with LDV (2 targets per plate, for all 7 plates; see Figure 2), 3 hammer hits were applied in *z*-axis at the centroid of the side face of the top plate of the structure to obtain the corresponding average force-response spectra.

A driving point measurement, i.e., an input–output set of data obtained from spatially coincident and coaxial force and response sensors, is required in EMA to obtain mass scaled mode shapes (and determine modal mass). However, in many practical applications this is impossible to realise due to physical constraints. Hence, an alternative sensor location needs to be used. In this study, the driving point measurement relied on the response sensors located around the perimeter of the top plate, as discussed in Section 2.2 and shown in Figure 2b,c. The difference in force and response sensors’ collocation is in this case acceptable since the high stiffness of the plate, together with uniform mass distribution, caused its lowest natural frequencies (at close to 1 kHz as estimated from a finite element model of a plate set in SAP2000) to be much higher than those of the frame, which were of interest. The coaxial alignment of the response sensor with the force sensor, apart from the skills of the impact hammer operator, relied on either the response sensor mounting arrangement and/or the transform of coordinates. A quality check was performed to verify this procedure, by inspecting the driving point frequency response function (FRF) magnitudes in which all resonance peaks, without exceptions, should be separated by antiresonance dips (with the corresponding abrupt shifts in the FRF phase). This is opposed to the transfer point FRF, obtained from non-collocated sensors between which there is differential motion, for which antiresonances do not need to occur. This was indeed the case, for which some supporting evidence is provided in Section 3.2.

The hammer signal was recorded together with the signals from accelerometers and LDV by their respective data acquisition systems, but not with the signals from other MCS. Therefore, the following signal time-alignment procedure was applied to be able to perform EMA using image-based MCS relying on finding a match between *z*-axis accelerometer signal from the top plate and spatially correspondent signals from those MCS. The signals were first up-sampled to a common frequency of 640 Hz. The acceleration and displacement signals were integrated and differentiated, respectively, to obtain velocity signals. The fourth-order two-way Butterworth band-pass filter with cut-off frequencies at 1 Hz and 20 Hz was applied throughout this process to minimise the errors associated with these numerical operations. The least-square error method was used to find the time lag based on the first 10 s of the acceleration signal measured after the impulse excitation, corresponding to the parts of response signals with the highest signal-to-noise ratio. The signals from image-based MCS were then time-aligned with the signals from accelerometers and the instrumented hammer, and truncated to match their length.

Siemens LMS SCADAS Mobile system was used for data acquisition from the accelerometers and the hammer. The hammer data were simultaneously logged by Polytec system to obtain a force signal time-synchronised with LDV signals.

The modal identification was performed using poly-reference least squares complex frequency domain algorithm [22,43,44,45] implemented in Siemens LMS Test.Lab™ 18.2 software under the name PolyMAX. PolyMAX has been shown to offer considerable advantages in terms of ease of use, performance speed, and reduced operator judgment dependency towards delivering high quality modal parameter estimations, even on complex data (e.g., noisy data from systems with high damping, high order, and closely spaced modes) [43]. It compares favourably with current best-of-class commercially available EMA techniques [44,46]. H1 estimator was used in the calculation of FRFs to reduce the effect of uncorrelated noise in the structural response signals. The results of modal analysis are presented in Section 3.2.

### 2.6. Initial Displacement Testing and Analysis

The initial displacement tests were conducted to assess the measurement accuracy at various motion amplitudes and the influence of the CGC angle of incidence on motion reconstruction with ArUco. The top plate was pulled approximately 5 mm away from its resting position in *z*-axis direction and then released. All optical MCS were simultaneously recording motion of the top plate of the frame for one minute at the time. The position and orientation of CGC was changed between tests, such that it was facing the side of the frame with ArUco markers at angles of 0, 15, and 30 degrees. The measurement point for LDV was chosen at the centroid of the side of the top plate facing the laser heads. The results of initial displacement tests are presented in Section 3.3.

## 3. Results and Discussion

### 3.1. ArUco Pose Accuracy Results

**Figure 6:**
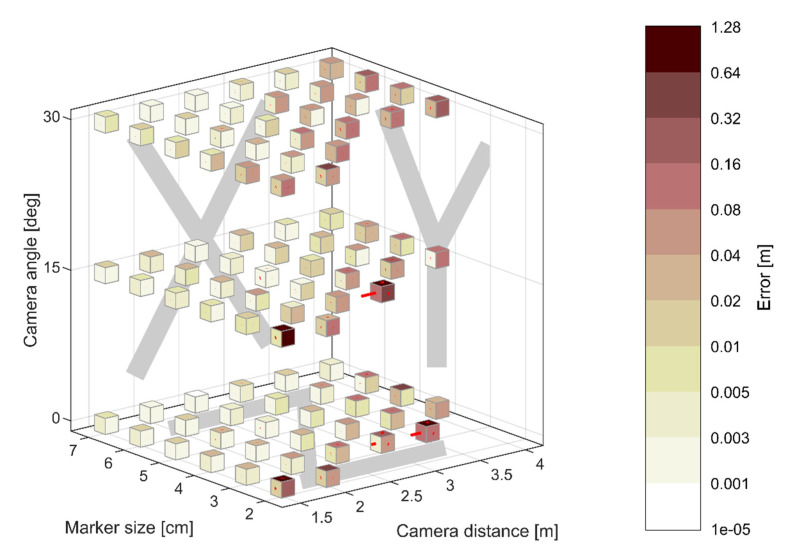
ArUco pose accuracy for various camera and markers arrangements in terms of mean and RMS distance error denoted by the colours of the cubes’ faces and the length of lines projecting out of the cube faces, respectively. The information associated with each face of the cube corresponds to the measurement along the axis denoted within the parallel backdrop plane, as defined in Figure 5.

The error estimates are plotted in Figure 6 for all camera and markers arrangements and for all measured orthogonal directions. The error is expressed as mean magnitude, denoted by colour coding of the faces of the cubes, and RMS values, denoted by the length of the red lines projecting out from the cubes’ faces. The information associated with each face of the cube corresponds to the measurement along the axis denoted within the parallel backdrop plane of Figure 6, as defined in Figure 5. The quality of reconstruction diminishes with increased distance of the camera from the frame, increased camera angle and decreased size of the markers. The same relationship is observed for the stability of reconstruction, expressed in terms of RMS error, which is associated with the measurement noise. No estimates were obtained for the cubes missing from the grid (see Section 2.4) for which the system arrangement was found inadequate for pose reconstruction. The least accuracy was obtained for the measurements in *z*-axis, as defined in Figure 5, which is in line with the results presented in Popescu et al. [34]. This is because ArUco markers are planar structures and the measurements along that axis, corresponding to the depth of the image, predominantly rely on the identification of their scale rather than displacement within the image plane. Any deviation of the camera angle from 0 degrees will amplify the scale difference between markers hence lead to an increased reconstruction error.

An appropriate camera and markers arrangement was specified considering a compromise between accuracy and applicability, in particular the availability of the area on the faces of the plates. Therefore, 5 × 5 cm ArUco markers were chosen and the CGC was placed 1.5 m away from the structure at a zero-degree angle of incidence (i.e., facing straight on the side of the frame).

### 3.2. Modal Testing Results

**Figure 7:**
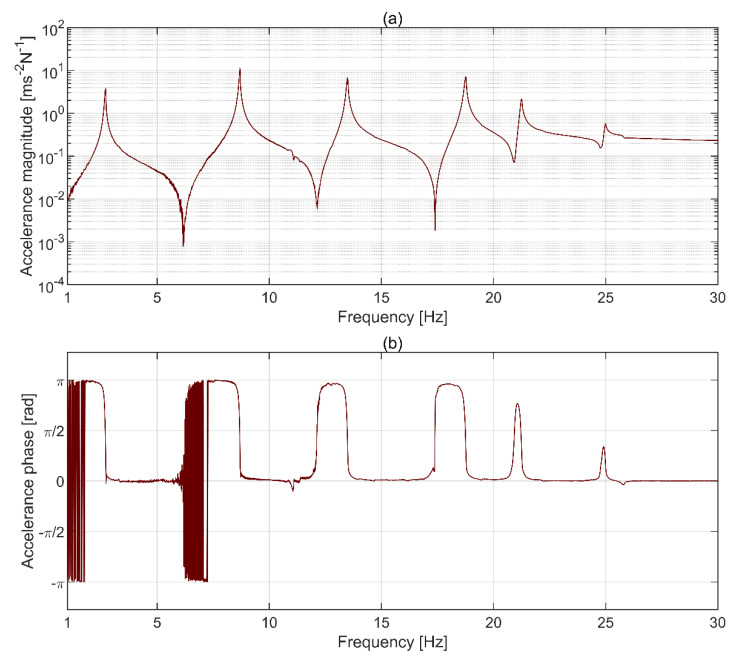

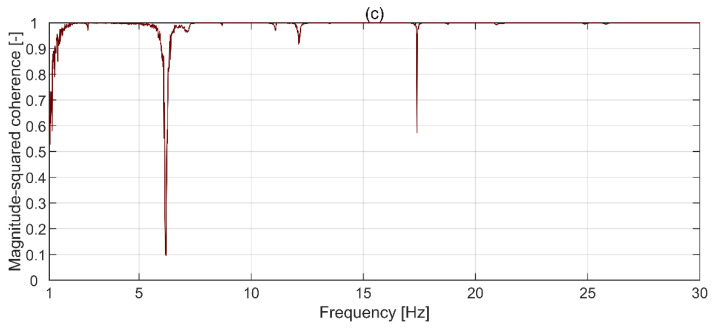
Driving point frequency response function (FRF) (**a**) magnitude, (**b**) phase, and (**c**) coherence for the accelerometer mounted at the top plate.

The driving point FRF obtained from an accelerometer mounted at the top plate is shown in Figure 7. Six resonant peaks in Figure 7a correspond to the phase of π/2 in Figure 7b, except for the last peak, close to 25 Hz, which has relatively small amplitude. Each pair of resonant peaks is separated by an antiresonance dip, which is to be expected for driving point FRF. The correlation between the force input and the structural response measured with accelerometers, shown in terms of the magnitude-squared coherence in Figure 7c, is very good for all resonant peaks and reduces for antiresonance dips. This is to be expected since any measured response at the antiresonances, and in theory there should be none, is then dominated by uncorrelated signals, e.g., sensor noise. Taken together, the results presented in Figure 7 support the assumptions made in dispersing sensors around the plates, as discussed in Section 2.5, and show that the direct point FRF used to scale the mode shapes is reliable.

**Figure 8:**
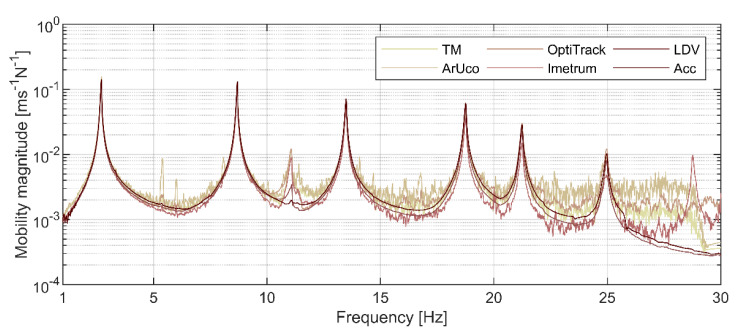
Averaged magnitudes of mobility FRF.

The magnitudes of FRF, averaged over all measurement points and expressed in terms of mobility, are shown in Figure 8. Mobility was chosen to reconcile measurements from MCS detecting displacement (for all image-based optical systems), velocity (for LDV) and acceleration (for accelerometers). The FRF is an average over eight windows, each containing data from a single hammer impulse test, apart from LDV for which three windows were used for each measurement point (see Section 2.5). Six well-separated dominant peaks, having magnitudes above 4 × 10^−3^ ms^−1^ N^−1^, are visible for accelerometry and LDV below the frequency of circa 25 Hz, marking the Nyquist frequency for Imetrum. These peaks correspond to the lightly-damped and well-separated *z*-axis translational modes constituting the focus of this study, hereafter denoted as mode 1 to 6. Although the behaviour of the frame above 25 Hz is not of interest, Figure 8 presents data up to 30 Hz to include the spectra roll-off for mode 6. A less defined peak is visible at frequency of around 11 Hz which was identified as a torsional mode using accelerometry (i.e., by finding significant common frequency components in spectra of x- and *z*-axis measurements), LDV and OptiTrack, each system intrinsically capable of providing 3D motion data. Mode 6 was not recovered by both CGC-based systems, and mode 5 was not recovered by ArUco. The peak in OptiTrack data at 28.8 Hz was not identified as a mode.

A visual inspection indicates the results from accelerometry and LDV to be seemingly compatible and relatively free from noise, which affects the performance of all other systems. The CGC-based systems are particularly prone to this problem, which is reinforced by their generally lower sensitivity.

There are slight differences in the peak amplitudes between MCS which could be attributed to systems’ specification, but also the spatial origin of data. This factor is associated with the systems’ operational requirements, and the requirement of having to accommodate numerous markers within each plate, as shown in Figure 2.

A complete set of numerical values for the identified modal parameters is given in Table 2. The values in brackets represent the percentage errors relative to the measurement with accelerometers.

**Figure 9:**
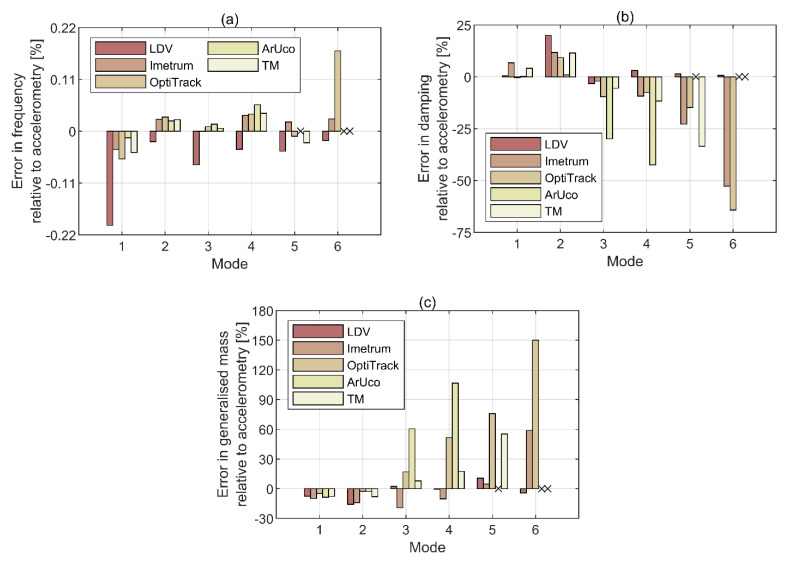
Errors in modal parameters relative to accelerometry in terms of (**a**) modal frequency, (**b**) damping, and (**c**) generalised mass. The data not recovered by ArUco and template matching systems are represented by crosses.

For better readability, the errors in modal parameter estimation are presented in a graphical way in Figure 9. The data not recovered by ArUco and template matching are indicated with crosses. As could be expected, the accuracy of frequency reconstruction presented in Figure 9a is overall high with the error magnitude below 0.2%. The discrepancy between results can be mainly attributed to the various sampling rates used by MCS, as stated in Table 1, hence various resolution of data in the frequency domain. The higher frequency resolution for LDV relative to accelerometry, imposed by the system requirements (i.e., the lowest sampling rate available in the LDV proprietary software), is the most likely reason for LDV underestimating modal frequencies across the whole range.

The identified damping levels are representative of those measured on bridges, chimneys, and steel masts and towers [24,47,48,49,50,51].

**Figure 10:**
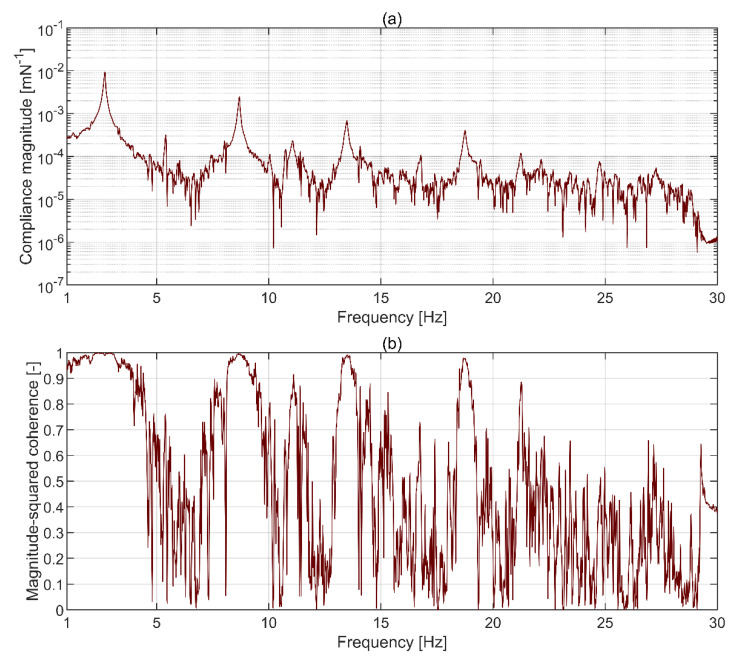
(**a**) Magnitude of compliance FRF and (**b**) corresponding magnitude-squared coherence for measurements with ArUco.

The accuracy of damping and generalised mass reconstruction, presented in Figure 9b,c, respectively, shows high variability between modes and within each mode above the 2nd mode. As could be expected from the system specifications, the highest overall accuracy relative to accelerometry is recorded for LDV. The relatively high difference in damping estimates for mode 2 is probably also related to the different ability of the instrumentation systems to capture the neighbouring torsional mode at approximately 11 Hz, as seen in Figure 8. In this particular case, the results obtained for LDV are more reliable. For all MCS based on image processing, the accuracy diminishes with the mode rank. The bias error was minimised by choosing the measurement duration allowing signals to decay to values below observable, considering the sensitivity of MCS, and using an exponential weighting window on both force and response measurements, hence minimising leakage. The random error in FRF obtained with H1 estimator, assuming no extraneous noise on the hammer force input (see Figure 3), provided by the formula given in Bendat and Piersol [52] is negligibly small for the recovered modes. Therefore, this systematic behaviour should be attributed to insufficient MCS sensitivity and relatively high noise levels obtained by taking the average from only eight windows. This can be understood by closer inspection of the magnitude of compliance FRF for ArUco system and the corresponding magnitude-squared coherence shown in Figure 10. The data were obtained by taking an average over eight windows, each containing data from a single hammer impulse test, and by applying an exponential window on both signals with the decay constant of −4, thus ensuring the window reduces to below 2% of its initial value of unity at the end of the record. The compliance FRF was chosen in this case as it relies on the displacement signal, which is native to ArUco. Four distinctive peaks are visible in the spectrum of compliance FRF magnitude above 4 × 10^−4^ mN^−1^ in Figure 10a, which correspond to the four lowest translational modes in *z*-axis of the frame. The peaks for mode 5 and 6 are hardly visible in the spectrum and mostly buried in the uncorrelated noise having magnitude reaching up to approximately 7 × 10^−5^ mN^−1^ within the corresponding frequency band. As shown in Figure 10b, the coherence is acceptable for the first four modes, but drops to unacceptable levels for frequencies associated with mode 5 and 6, for which the magnitude FRF in Figure 10a is heavily distorted between resonant peaks. In comparison, the mobility FRF magnitude for accelerometers in Figure 8, from data sampled at a similar rate to CGC systems (see Table 1), is very clear and there is no distortion throughout the whole frequency bandwidth.

Interestingly, the performance of Imetrum and template matching in terms of modal frequency, damping and generalised mass is comparable up to the 4th mode at circa 18.73 Hz. The two MCS relied on different fiducial markers and Imetrum used two cameras whilst template matching used only one camera. Furthermore, the results from Imetrum were averaged over two markers tracked within the top plate, whilst a single marker was tracked by template matching. Beyond the 4th mode, all image-based MCS yield little accuracy in terms of damping and generalised mass reconstruction.

**Figure 11:**
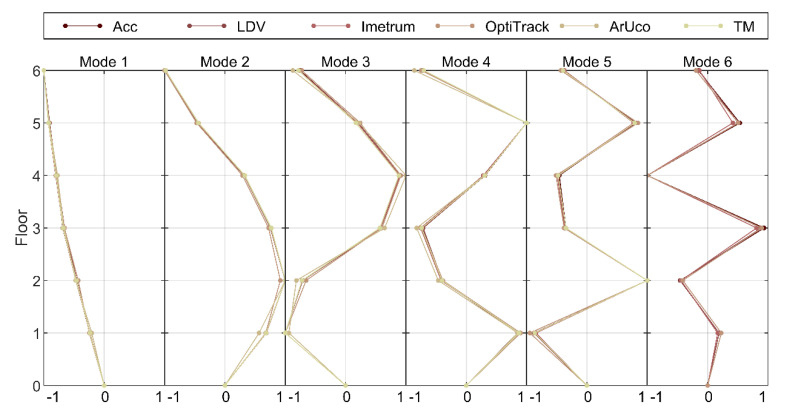
The first six translational mode shapes of the frame in *z*-axis.

Another reason for the deficiency in reconstruction of mode 5 and 6 by CGC-based MCS is revealed when considering relatively low response levels for these modes at the top plate, as shown in Figure 8, together with the mode shapes, shown in Figure 11. For clarity of presentation, the mode shapes in Figure 11 are normalised to the maximum magnitude of unity. Although the relative resolution of mode shapes coarsens with the increasing mode shape rank, the relative movement at the measurement points is what could be expected from the type of tested structure when idealised as a lumped-mass model. The match between mode shapes recovered with different MCS seems generally good. However, the modal displacement at the top plate, coinciding with the point of application of the hammer force, is relatively small for mode 5 and 6. This means that the energy transmitted to these modes, bearing in mind the force level was similar across the whole frequency range of interest, could have been insufficient to excite them beyond the noise floor of image-based MCS. This also explains low coherence for mode 5 and 6 in Figure 10b.

**Figure 12:**
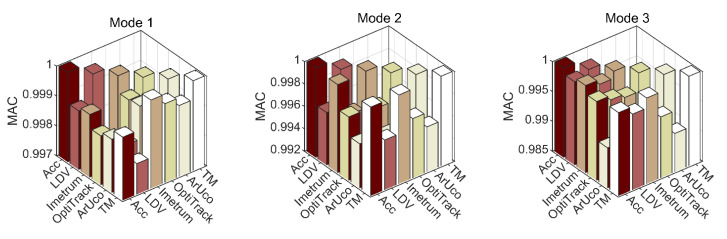

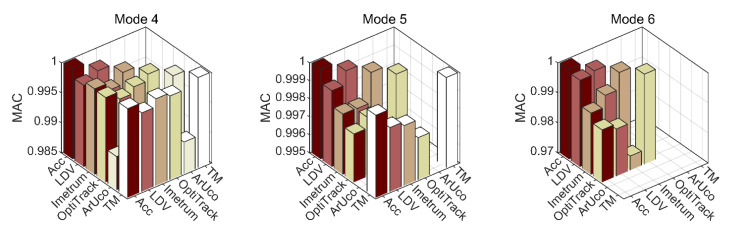
Modal assurance criterion (MAC) based on eigenvectors obtained from data recorded with all motion capture systems (MCS) for the first six translational mode shapes. The scale of the vertical axis is adjusted such as to contain all available results.

Figure 12 presents modal assurance criterion (MAC) [53,54] calculated to quantify the correlation between mode shapes for the first six translational modes. Overall, MAC obtained against accelerometry for all MCS is above 0.987 which, for all practical purposes, can be considered satisfactory. The most consistent agreement with accelerometry for mode 6 is found for LDV. The MAC for mode 6 for the other two systems which recovered this mode shape, i.e., Imetrum and OptiTrack, deteriorates significantly relative to the MAC for lower rank modes.

**Figure 13:**
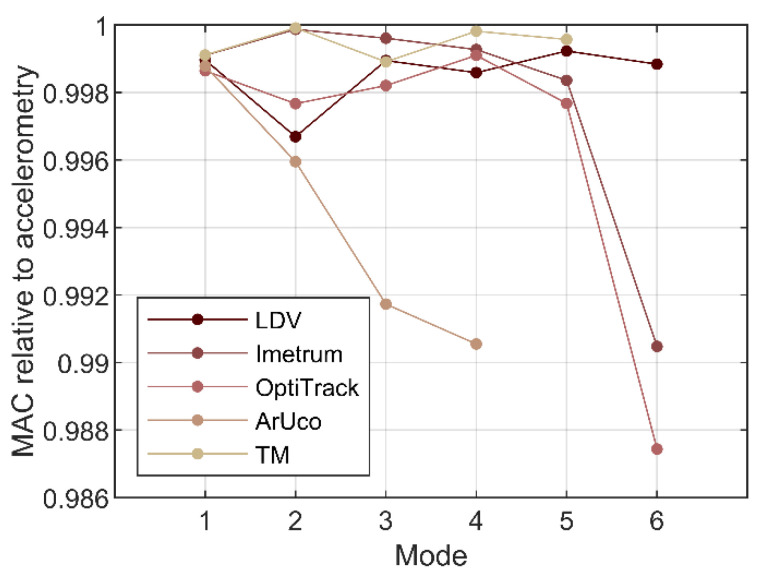
Modal assurance criterion (MAC) for all optical MCS relative to accelerometry.

The correlation of mode shapes obtained with ArUco with those obtained with accelerometry deteriorates consistently with the mode shape rank. This is clearly visible in Figure 13, which is a snapshot of the results presented in Figure 12.

In conclusion to this section, the benchmark estimates of modal parameters are assumed here to come from accelerometry and LDV, since they give corresponding results in terms of the modal frequency, damping and generalised mass, as seen in Table 2 and Figure 9, and the mode shape, as seen Figure 11 and Figure 12. This is the case for all translational modes considered in this study, with the exception of mode 2 of which identification is affected by the proximity of a torsional mode. All image-based MCS give corresponding results for the lowest modes, but the error between estimates of modal parameters increases with the mode rank.

The errors in modal parameters obtained with image-based MCS are not out of range with results reported from an exercise aiming to assess the consistency in modal identification of a structure moved between twelve European laboratories [55]. The variability in the estimated modal frequency, damping and generalised mass, obtained from accelerometry, was within 4%, 30%, and 10%, respectively.

The performance of image-based commercial MCS—Vicon (similar to OptiTrack used herein), based on three cameras and light-reflective markers, was compared to laser displacement sensors (LDS) in Park et al. [56]. The measurements were taken from a scaled model of a pylon subjected to uniform wind flow within a wind tunnel. The frequency and damping estimates obtained from LDS for the first translational mode were 6.93 Hz and 0.359%, respectively. The corresponding estimates from Vicon were within 0.3% and 177%, respectively. In a subsequent study [24], the performance of Vicon, consisting of four cameras, was compared to accelerometry and LDS. The behaviour of a scaled model of a 3D building frame having three storeys (rather than six storeys as in the model used herein) and stiffened with bracing in one lateral direction to enforce dominant vibrations in the other lateral direction, in response to an initial displacement, was of interest. There were no errors, up to two decimal places, in the frequencies of three translational modes between 1.07 Hz and 5.15 Hz and the error in modal damping, expected to fall within the range of 0.2% to 0.84%, was within approximately 2%. However, none of these studies reported modal mass estimates nor conducted EMA.

An alternative way to present the results obtained herein would be to normalise mode shapes by the modal mass rather than the maximum amplitude at the measurement points. The MAC obtained from such normalised mode shapes would remain the same yet the modes themselves would carry the same error with the reported modal mass. However, this would mask the fact that the relative movement of measurement points on the tested structure is consistent in scale between all deployed instrumentation systems.

### 3.3. Initial Displacement Testing Results

Having established that image-based MCS provide modal parameters compatible with benchmark estimates from accelerometry and LDV for mode 1 and 2, the focus in this section is on these two modes only. The LDV data were used herein as a benchmark to avoid numerical errors associated with double-integration of the acceleration data and to account for better performance of this system in capturing characteristics of mode 2. All MCS data were up-sampled to a common frequency of 3 kHz for precise time alignment.

#### 3.3.1. Vibration Amplitude

**Figure 14:**
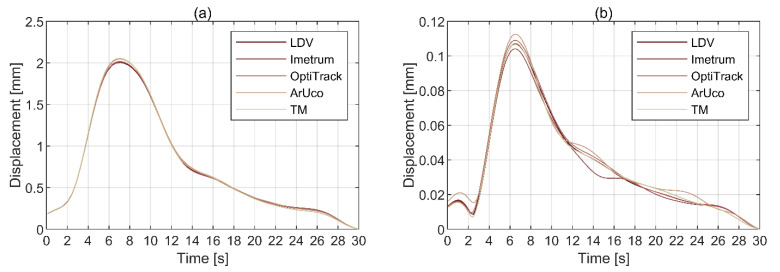
Envelopes of the amplitudes of peak displacement for (**a**) mode 1 and (**b**) mode 2.

The envelopes of the amplitude of peak displacement identified within each vibration cycle for the first two modes are shown in Figure 14. The data were obtained by applying a two-way fourth-order Butterworth band-pass filter with cut off frequencies ±0.15 Hz from the identified modal frequencies. The ArUco signal used in the comparison was recorded at 0 degrees CGC incidence angle relative to the frame (see Figure 2). It can be seen that all four image-based MCS are capable of measuring dynamic displacements with sub-millimetre (and sub-pixel) accuracy. Visual inspection indicates that the motion of the top plate in mode 1, shown in Figure 14a, is recovered with good accuracy for all image-based MCS relative to the benchmark LDV data. For mode 2, the signals from Imetrum and ArUco, shown in Figure 14b, diverge from the benchmark LDV data for amplitudes below 0.05 mm. However, it needs to be noted that the motion amplitudes recorded for mode 1 and 2 differ by more than an order of magnitude. The RMS errors in peak displacement amplitudes relative to LDV are given in Table 3.

The RMS errors are generally small for all MCS, falling below 0.02 mm. The best match with LDV is found for OptiTrack. Although the accuracy generally improves with the number of cameras in MCS (see Table 1), overall, the performance of MCS relying on a single CGC either matches that of multi-camera systems or is not out of range by much.

#### 3.3.2. Influence of the Angle of Incidence of CGC

**Figure 15:**
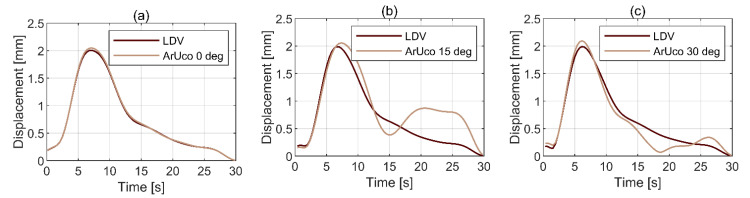
ArUco reconstruction of motion amplitude for mode 1 depending on the CGC angle of incidence: (**a**) 0 degrees, (**b**) 15 degrees, (**c**) 30 degrees.

A dedicated set of tests was conducted to establish the influence of the CGC angle of incidence on the accuracy of vibration measurement with ArUco. The CGC was set at three angles of incidence: 0, 15, and 30 degrees. The results of these tests, together with the benchmark data from LDV, are presented in Figure 15a–c, respectively. It can be seen that all angles other than 0 degrees generate significant errors. This is predominantly caused by the motion component out of plane relative to the camera and is consistent with the results of static pose reconstruction discussed in Section 3.1.

### 3.4. Limitations of This Study and Deployment Considerations on In Situ Structures

The way various systems were set up is of course specific to the conducted tests. Although every effort was made to make informed decisions and apply best judgement, it is possible that the systems were not operated at their optima. For example, the choice of fiducial markers and, to some extent, their location on the structure, as explicitly studied elsewhere [7], was often arbitrary, dictated by suggestions reported in the literature and authors’ own experience.

There are certain limitations and difficulties in using optical MCS beyond those related to their intrinsic design, which, ultimately, can lead to the reduced quality measurements in structural vibration monitoring. These factors include uncertainty in system calibration, environmental effects, and deployment difficulties [1]. For example, light intensity fluctuations during monitoring, e.g., those associated with alternating cloud coverage, and light refraction can lead to drift and sudden discontinuities in the measured signals. These problems can be minimised in post processing, e.g., with zero-normalised versions of certain correlation criteria for template matching method [57]. Changes in atmospheric pressure and wind speed can be prevalent [5]. Camera instability or drift associated with settlement can be reduced by ensuring rigid camera mounting [58] or using an auxiliary reference point within the captured image, stationary in the absolute reference frame, and subtracting its motion from the measured structural displacement [6]. Camera vibration can also be compensated for, to some extent, by incorporating an accelerometer recording camera motion for use in post processing [59]. However, most of these challenges, which are often coincident, cannot be completely eliminated during in situ investigations on outdoor structures. Therefore, to obtain a benchmark performance of optical structural vibration monitoring systems, a controlled environment is required, which shelters them from the elements. This was achieved in the current study.

## 4. Conclusions

The performance of five optical motion capture systems (MCS) in experimental modal analysis (EMA) is investigated in this study, based on the data obtained from a scaled model of a frame of a 3D building. This includes Polytec laser Doppler vibrometer—LDV, two commercial multi-camera systems—OptiTrack and Imetrum, and two open-source systems utilising a single consumer-grade camera (CGC) based on feature- and area-based template matching—ArUco and TM, respectively. The results obtained with accelerometry are used as a validating benchmark. A complete set of modal parameters is considered, including modal frequency, mode shape, modal damping and modal (generalised) mass. To the best of authors’ knowledge, this experimental study constitutes the broadest to date comparative effort towards understanding the performance of optical MCS in EMA.

Dictated by the specification of some of the deployed MCS, the translational response of the frame associated with the weak axis of the columns is of interest, up to the frequency of 25 Hz. Six translational modes are identified by all systems, apart from ArUco which could not recover mode 5 at 21.2 Hz and mode 6 at 24.9 Hz, and TM which could not recover mode 6. The maximum discrepancy in modal frequency is below 0.2% and can be explained by different sampling rates inherent to the deployed MCS. The modal assurance criterion calculated for the empirical mode shapes does not fall below 0.987.

The modal parameters derived from LDV are in excellent agreement with accelerometry for all considered modes. This is apart from the damping for mode 2, of which reconstruction is affected by the neighbouring torsional mode, which is identified with greater accuracy by LDV rather than accelerometry. All image-based MCS, i.e., OptiTrack, Imetrum, ArUco and TM, can measure the displacement with sub-millimetre and sub-pixel accuracy. A good match with a benchmark measurement from LDV is found for amplitudes down to 0.05 mm. Considering all image-based MCS, the approximate maximum error magnitude in modal damping and mass is, respectively, 7% and 10% for mode 1 at 2.7 Hz, and 12% and 14% for mode 2 at 8.7 Hz. All modal parameters for mode 3 at 13.5 Hz and mode 4 at 18.7 Hz are recovered reasonably well by all systems apart from ArUco, with the maximum error in modal damping and mass below 12% and 20%, respectively. Another exception is Imetrum for which the error in modal mass reconstruction for mode 4 is above 50%. The performance of all image-based MCS in the reconstruction of modal damping and mass for mode 5 and 6 is unsatisfactory. This is caused by a combination of factors, most notably low response levels at these modes, relatively low sensitivity and high noise floor of these MCS, and mode 6 being close to the Nyquist frequency.

ArUco system, relying on feature-based template matching, performs best with the CGC positioned at 0 degrees angle of incidence. This is found in static pose reconstruction tests and in vibration tests. The more the angle of incidence deviates from 0 degrees, the higher measurement error. When implemented with CGC at its preferable orientation, an area-based template matching algorithm can match the performance of Imetrum, relying on two cameras, in the lowest four modes. The error magnitude in modal damping and mass is then below 20%.

In general, image-based MCS do not match the performance of conventional accelerometers nor LDV in terms of sensitivity and noise levels. Therefore, for maximum efficiency, the organisation of instrumentation systems during the tests and the testing protocols needs to be carefully considered to ensure all modes of interest are sufficiently excited by the force source and can be recovered in modal analysis. Since it is always a good practice to conduct a survey to identify a suitable arrangement of instrumentation prior to any modal test, the penalty associated with this task may not be significant.

Overall, the presented results suggest that MCS based on a single consumer-grade camera and open-source tracking algorithm is capable of providing data enabling EMA to be conducted when frequencies of interest fall below approximately 20 Hz, which is the case for many civil engineering structures. The main advantage of a system of this type is in the ease of deployment and economy—the cost, which can be limited to hardware only, is a small fraction of that associated with commercial systems. Therefore, the outcomes of this study encourage further efforts in exploring the performance and optimisation, e.g., through hybrid solutions, of image-based optical motion capture systems in the context of fully detailed EMA, rather than the identification of modal frequencies and mode shapes only.

## Figures and Tables

**Table 1:** Basic specification of the motion capture systems used in this study.

System	Sensor	Quantity	Sampling Frequency and Resolution	Distance Relative to the Markers	Fiducial Marker
Polytec LDV	PSV-500-HM	3	200 Hz and 0.02 µm/s	3 m	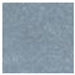
OptiTrack	Prime 13 with 5.5 mm lens	8	120 fps at 1.3 MP	1–3 m	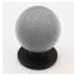
Imetrum	Manta G-223 B NIR with 16 mm lens	2	50 fps at 2 MP	3 m	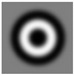
Accelerometers	PCB 356 A16 PCB 333 B30	5 2	64 Hz at 100 mV/g 64 Hz at 100 mV/g	Direct contact Direct contact	N/A
CGC (used with feature-based ArUco and area-based template matching)	Cannon EOS 200 D with DIGIC 7 processor and 20 mm Canon lens	1	59.94 fps at 24.2 MP	1.5 m	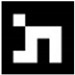

**Table 2:** Modal parameters of the structure identified with various instrumentation systems for the first six translational modes in *z*-axis.

	**Modal Frequency [Hz]**					
	**Mode 1**	**Mode 2**	**Mode 3**	**Mode 4**	**Mode 5**	**Mode 6**
**Accelerometry**	2.681 (N/A)	8.673 (N/A)	13.471 (N/A)	18.730 (N/A)	21.223 (N/A)	24.943 (N/A)
**LDV**	2.676 (−0.20%)	8.671 (−0.02%)	13.462 (−0.07%)	18.723 (−0.04%)	21.214 (−0.04%)	24.938 (−0.02)
**OptiTrack**	2.680 (−0.04%)	8.675 (0.02%)	13.471 (0.00%)	18.737 (0.03%)	21.227 (0.02%)	24.949 (0.03%)
**Imetrum**	2.680 (−0.06%)	8.676 (0.03%)	13.473(0.01%)	18.737 (0.04%)	21.221 (−0.01%)	24.985 (0.17%)
**ArUco**	2.681 (−0.01%)	8.675 (0.02%)	13.473 (0.01%)	18.741 (0.06%)	N/A	N/A
**Template matching**	2.680 (−0.05%)	8.675 (0.02%)	13.472 (0.01%)	18.738 (0.04%)	21.218 (−0.02%)	N/A
	**Modal damping ratio [%]**					
	**Mode 1**	**Mode 2**	**Mode 3**	**Mode 4**	**Mode 5**	**Mode 6**
**Accelerometry**	0.706 (N/A)	0.224 (N/A)	0.251 (N/A)	0.227 (N/A)	0.238 (N/A)	0.233 (N/A)
**LDV**	0.710 (0.54%)	0.269 (20.14%)	0.243 (−3.24%)	0.234 (3.05%)	0.241 (1.46%)	0.235 (0.70%)
**OptiTrack**	0.754 (6.79%)	0.250 (11.77%)	0.246 (−2.03%)	0.206 (−9.25%)	0.184 (−22.77%)	0.110 (−52.73%)
**Imetrum**	0.704 (−0.31%)	0.245 (9.29%)	0.227 (−9.59%)	0.210 (−7.52%)	0.203 (−14.76%)	0.083 (−64.23%)
**ArUco**	0.707 (0.21%)	0.226 (0.93%)	0.176 (−29.95%)	0.130 (−42.47%)	N/A	N/A
**Template matching**	0.735 (4.13%)	0.249 (11.51%)	0.237 (−5.47%)	0.200 (−11.68%)	0.158 (−33.54%)	N/A
	**Generalised mass [kg]**					
	**Mode 1**	**Mode 2**	**Mode 3**	**Mode 4**	**Mode 5**	**Mode 6**
**Accelerometry**	19.55 (N/A)	20.74 (N/A)	17.71 (N/A)	15.55 (N/A)	13.80 (N/A)	10.78 (N/A)
**LDV**	18.05 (−7.66%)	17.47 (−15.78%)	18.11 (2.27%)	15.44 (−0.72%)	15.26 (10.59%)	10.31 (−4.38%)
**OptiTrack**	17.57 (−10.11%)	17.83 (−14.04%)	14.27 (−19.41%)	13.95 (−10.26%)	14.45 (4.71%)	17.13 (58.92%)
**Imetrum**	18.58 (−4.93%)	20.18 (−2.72%)	20.72 (16.98%)	23.58 (51.65%)	24.25 (75.76%)	26.95 (149.99%)
**ArUco**	17.85 (−8.65%)	20.13 (−2.92%)	28.42 (60.50%)	32.12 (106.6%)	N/A	N/A
**Template matching**	18.02 (−7.83%)	19.06 (−8.07%)	19.11 (7.93%)	18.28 (17.57%)	21.43 (55.34%)	N/A

**Table 3:** RMS errors in the amplitudes of peak displacement for mode 1 and mode 2 relative to LDV.

MCS	Number of Cameras	Mode 1	Mode 2
		RMS Error [mm]	RMS Error [mm]
OptiTrack	8	6.74 × 10^−3^	1.23 × 10^−3^
Imetrum	2	7.15 × 10^−3^	2.61 × 10^−3^
ArUco	1	1.74 × 10^−2^	3.12 × 10^−3^
Template matching	1	1.98 × 10^−2^	1.34 × 10^−3^

## Data Availability

Available upon request from the corresponding author.
